# Transient Atrial Fibrillation After Epinephrine Use for Anaphylaxis

**DOI:** 10.7759/cureus.32230

**Published:** 2022-12-05

**Authors:** Park Dahye, Asad Khan

**Affiliations:** 1 School of Medicine, University of Alabama at Birmingham School of Medicine, Birmingham, USA; 2 Internal Medicine, Hospitalist Service of Southeast Alabama, Montgomery, USA

**Keywords:** case report, adverse events, atrial fibrillation, epinephrine, anaphylaxis

## Abstract

Anaphylaxis is defined as a generalized or systemic hypersensitivity reaction that occurs after exposure to certain antigens. Clinical presentations vary but life-threatening symptoms include upper and lower airway obstruction, which requires immediate treatment. Current standard treatment for anaphylactic shock includes epinephrine and volume resuscitation. Cardiac symptoms after epinephrine treatment in a patient with no prior cardiac history are often not suspected. In this case report, we present an unusual presentation of transient atrial fibrillation after an insect sting anaphylaxis and treatment with intramuscular (IM) and intravenous (IV) epinephrine.

## Introduction

Atrial fibrillation (AF) is the most common arrhythmia, mainly associated with cardiovascular disorders, including hypertension and coronary heart disease. Furthermore, certain drugs that stimulate the adrenergic or vagal system may induce acute AF in patients without prior cardiac history [1]. Epinephrine, also known as adrenaline, is the main drug of choice for the treatment of anaphylaxis [2]. Epinephrine stimulates the adrenergic system but epinephrine-induced AF is a rare phenomenon. In this case report, we present an unusual presentation of transient AF after an insect sting anaphylaxis treated with intramuscular (IM) and intravenous (IV) epinephrine.

## Case presentation

A 57-year-old female with no known past medical history presented to the emergency department (ED) with an anaphylactic reaction, including shortness of breath, facial swelling, and a generalized rash all over her body after an insect sting on her left arm. The patient was given IM epinephrine twice, IV Benadryl 50 mg once, and IV fluids by the paramedics. Upon presentation, the patient was lethargic but easily aroused. Initial vital signs were a temperature of 95.3 F, blood pressure of 72/45 mmHg, and heart rate of 120. Chest X-ray showed no acute findings. The patient's oxygen saturation was 98% on 2 liters of oxygen on a nasal cannula. While in the ED, she continued to be hypotensive and hypothermic. The patient was given 0.5 IM epinephrine, an additional 2 liters of lactated Ringers boluses, IV Decadron, and Solu-Medrol. Furthermore, the patient received an IV epinephrine additive of 4 mg plus NS premix diluent 250 mL. Labs showed normal CBC, initial potassium of 2.7, lactic acid 4.1, and increased troponin of 4.44 to 675.31 ng/L.

**Figure 1:**
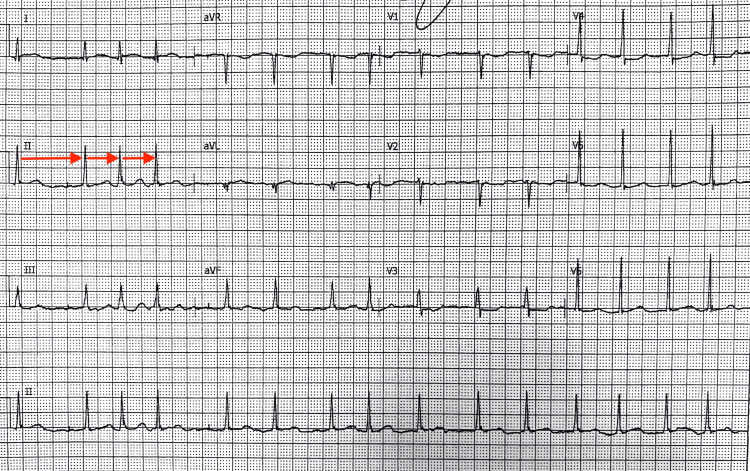
EKG after epinephrine administration showing no P waves and irregular distances between R and Rs (red arrows)

Thirty minutes after starting IV epinephrine additive 4 mg, the patient’s electrocardiogram (EKG) showed atrial fibrillation at a pulse rate of 92/m with non-ST elevation myocardial ischemia (NSTEMI) Type II (Figure 1). A few hours later, the patient was back in sinus rhythm. According to the cardiologist, the patient’s EKG showed non-diagnostic repolarization changes in addition to her echocardiogram (ECHO) showing elevated right atrial pressure, likely in the setting of bronchospasm related to anaphylaxis. Per the cardiologist, the patient’s transient AF was likely triggered by the sympathetic surge from exogenous epinephrine administration. Additionally, a coronary angiogram was not recommended during hospitalization due to no significant findings on the echocardiogram (ECHO) and EKG. The patient was not initiated on anticoagulation after discussing with cardiology given the transient AF.

**Figure 2:**
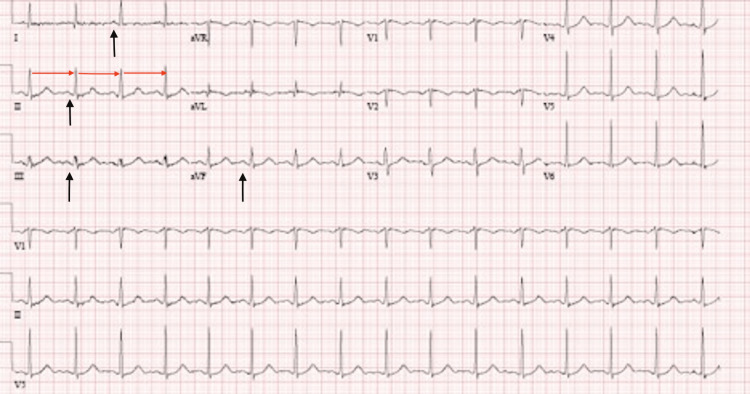
EKG at two days of hospitalization showing regular distances between R and Rs (red arrows) and P waves (black arrows)

The patient was admitted to the intensive care unit (ICU) for further management and was given continued IV epinephrine additive 4 mg plus NS premix diluent 250 mL. On day 2, IV epinephrine was discontinued due to recurrent urticaria and was eventually stopped on day 3. A repeat EKG done two days after admission (Figure 2) showed normal sinus rhythm, and no further episodes of AF were noted during her hospital course. The patient was discharged on day 3 with a prescription of prednisone 5 mg, Montelukast 10 mg once daily for 30 days, loratadine twice daily for 10 days, and famotidine 20 mg twice daily for 14 days. The patient reported no symptoms during the post-discharge phone call, and she refused any further outpatient cardiac evaluations.

## Discussion

In clinical practice, epinephrine-induced AF is a rare phenomenon. There has been one case report of acute AF after an epinephrine overdose [3]. In our anaphylactic patient with no prior cardiac history, the administration of IV epinephrine resulted in drug-induced AF.

AF is the most common cardiac arrhythmia. It is due to abnormal firing of the atrium that results in an unsynchronized rate and irregular rhythm, which can increase the risk of stroke [4]. An autonomic nervous system, including adrenergic and vagal stimulation or inhibition, can influence cardiac electrical conduction, resulting in irregular heart rate and rhythm. Therefore, certain drugs that affect atrial electrical properties or adrenergic or vagal stimulation may induce new-onset AF in patients without prior cardiac history [1].

Epinephrine is a sympathomimetic drug that primarily acts on adrenergic receptors. It is a drug of choice for anaphylaxis, as it induces vascular constriction and bronchodilation and increases myocardial contractility [5-6]. For acute anaphylaxis treatment, IM injection is the preferred route of administration due to the faster onset of action, followed by IV and subcutaneous epinephrine. The standard dose of epinephrine at any age is 0.01 mg/kg of a 1:1000 dilution per single dose, with a maximum dose of 0.5 mg. Repeated dosage of epinephrine may be administered at 5-15 minute intervals if the patient is unresponsive to the treatment [7]. Since epinephrine mimics the sympathetic nervous system, common side effects include tachycardia, hypertension, headache, anxiety, and palpitations. More serious adverse effects include angina, cardiac arrhythmias in patients with or without heart disease, and ventricular fibrillation [5]. To prevent any serious cardiac complications, the dosage of epinephrine must be carefully considered and administered.

The clinical finding of transient AF after IV epinephrine administration suggests drug-induced AF. Additionally, the activation of the sympathetic nervous system and coronary vasoconstriction associated with epinephrine use may have induced non-ST-elevation myocardial infarction (NSTEMI) type II in our patient. Despite any further treatment, the patient's new-onset AF converted into sinus rhythm during the hospital course. As shown in our case, medical teams providing care to patients with anaphylaxis should take into consideration the possibility of new-onset AF with epinephrine treatment. Cardiac monitoring should be performed before and after epinephrine administration to prevent any further complications. If a drug-induced AF is suspected in a patient, medication history should be reassessed and the suspected medication should be discontinued. Patients should be given an alternative treatment and repeat EKG and ECHO should be performed to rule out additional cardiac abnormalities. Furthermore, patients presenting with drug-induced AF should be reassessed for any recurrence of AF.

## Conclusions

Our case suggests that IV epinephrine treatment in patients with an anaphylactic reaction may result in drug-induced AF regardless of prior cardiac history. Physicians and medical staff providing care to patients with anaphylaxis should take into consideration the possibility of new-onset AF with epinephrine treatment. If a drug-induced AF is suspected, the suspected medication should be discontinued. Additional cardiac imaging and continuous cardiac monitoring will be helpful for further management. If AF remains chronic or recurs, proper treatment should be provided to the patient to reduce complications associated with AF.
